# Management of cesarean section in a patient with Fontan circulation: a case report of dramatic reduction of maternal oxygen consumption after delivery

**DOI:** 10.1186/s40981-020-00385-w

**Published:** 2020-10-04

**Authors:** Kazutomo Saito, Hiroaki Toyama, Atsushi Okamoto, Masanori Yamauchi

**Affiliations:** 1 Anesthesiology and Perioperative Medicine, Tohoku University Graduate School of Medicine, 2-1 Seiryomachi, Aoba-ku, Sendai, Miyagi 980-8575 Japan; 2 Department of Anesthesiology, Tohoku University Hospital, 1-1 Seiryomachi, Aoba-ku, Sendai, Miyagi 980-8575 Japan

**Keywords:** Adult congenital heart disease, Tricuspid atresia, Fontan circulation, Cesarean section, Central venous oxygen saturation, Oxygen consumption

## Abstract

**Background:**

The anesthetic management of cesarean sections in Fontan-palliated parturients requires strict hemodynamic control. However, patient management with central venous oxygen saturation (ScvO_2_) and oxygen consumption (VO_2_) has never been reported.

**Case presentation:**

A 30-year-old woman, who had received a total cavopulmonary connection for tricuspid atresia, was planned to undergo cesarean section at 38 weeks’ gestation. During combined spinal-epidural anesthesia, ScvO_2_ in addition to arterial pressure-based cardiac output (APCO) and central venous pressure (CVP) was monitored, and the change of VO_2_ was evaluated. After delivery, her APCO was almost unchanged. However, her ScvO_2_ increased dramatically from 42.1 to 67.3% and her CVP increased from 9 to 11 mm Hg. The calculated mean maternal VO_2_ changed from 443 to 295 mL/min.

**Conclusions:**

In a cesarean section for a Fontan-palliated parturient, ScvO_2_ dramatically increased and maternal VO_2_ decreased by more than 25% after delivery.

## Background

The long-term survival rate of patients with congenital heart disease (CHD) has been improved through progress in surgical techniques and multidisciplinary perioperative management methods [1, 2]. Accordingly, the number of women with CHD who experience pregnancy and delivery is increasing [3, 4]. Fontan physiology is characterized by a single ventricle maintaining systemic and pulmonary circulation. Since pulmonary circulation in Fontan-palliated patients is driven by central venous pressure (CVP), these patients require a higher than normal CVP. Consequently, they are less tolerant to volume overload [5] and thus require strict circulation management during anesthesia.

During pregnancy, dynamic physiological changes occur, including an increase in cardiac output, oxygen consumption, and intravascular volume, making both pregnancy and delivery challenging for these patients [6]. Previous reports have described the perioperative management of cesarean section in Fontan-palliated parturients [7–10]. However, hemodynamic monitoring of central venous oxygen saturation (ScvO_2_) and oxygen consumption (VO_2_) during this procedure has never been reported. Here, we present the first case of a cesarean section during which ScvO_2_, arterial pressure-based cardiac output (APCO), and CVP were monitored in a patient with Fontan circulation. Furthermore, we examined perioperative changes in maternal VO_2_. Our report will contribute to the understanding of the hemodynamic and VO_2_ changes that occur during cesarean delivery in patients with Fontan circulation.

## Case description

A female infant, diagnosed with tricuspid atresia (TA) type 1B (i.e., ventricular septal defect and pulmonary stenosis), underwent a balloon atrioseptostomy at 17 days of age and a Fontan operation at the age of 4 years. At age 12, she developed low percutaneous oxygen saturation (SpO_2_; 90%) and presented an exacerbation of symptoms, including cyanosis, clubbed fingers, and hepatomegaly. She underwent an extracardiac total cavopulmonary connection. Her postoperative CVP was 15 mm Hg. This procedure improved her New York Heart Association (NYHA) functional classification to class I. At 28 years old, her peak VO_2_, assessed by a cardiopulmonary exercise test, was 62.2% of that of healthy age-mates. Echocardiography indicated that her left ventricular ejection fraction (LVEF) was 64% without atrioventricular valvular regurgitation.

At age 30, she became pregnant and was referred to our hospital at 15 weeks’ gestation. Her usual anticoagulation therapy was changed from warfarin to aspirin because of the former’s teratogenic potential. A Holter electrocardiogram revealed a sinus rhythm of 91 beats/min without any episodes of arrhythmia. At 35 weeks’ gestation, she developed exertional dyspnea, followed by cyanosis (SpO_2_, 89% in room air), although she had an LVEF of 61% without left ventricular dilation. Her NYHA classification changed to class II. She was admitted to our hospital at 36 weeks’ gestation. Her blood hemoglobin decreased from 13.9 to 10.8 g/dL antenatally. Because her exercise capacity was 60% of that of healthy age-mates, a cesarean section was performed at 38 weeks’ gestation. Her anticoagulation therapy (intravenous unfractionated heparin, 10,000 U/day) was discontinued 6 h before surgery.

**Fig. 1:**
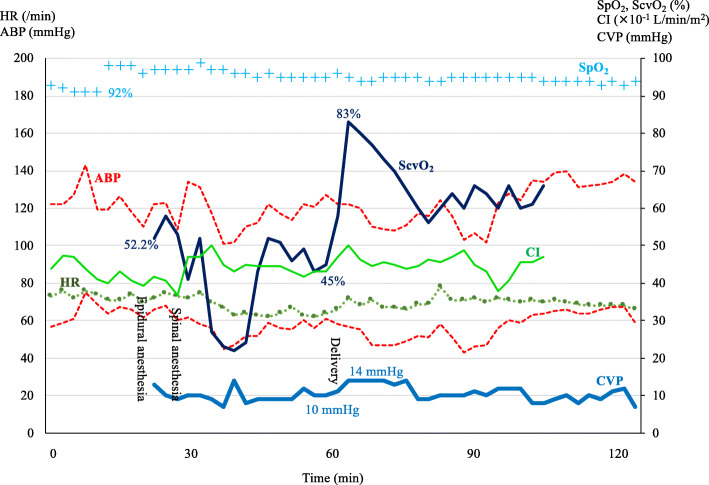
Trend of hemodynamic parameters during anesthesia. HR, heart rate; ABP, arterial blood pressure; SpO_2_, percutaneous oxygen saturation; ScvO_2_, central venous oxygen saturation; CI, cardiac index; CVP, central venous pressure

In the operating room, the patient’s electrocardiogram, SpO_2_, arterial blood pressure (ABP), and APCO were monitored via a left radial artery catheter (FloTrac Sensor^TM^; Edwards Lifesciences Corporation, Irvine, CA, USA). Additionally, a central venous catheter (PreSep Catheter^TM^; Edwards Lifesciences) was inserted via the right internal jugular vein to monitor CVP and ScvO_2_ continuously. The tip of the central venous catheter was located just cephalad to the connection between the superior vena cava and the right pulmonary artery by chest X-ray examination. On room air, the patient’s SpO_2_, CVP, and ScvO_2_ levels were 92% (PaO_2_, 69 mm Hg), 10 mm Hg, and 52.2%, respectively (Table 1). After the initiation of oxygen administration at 5 L/min, she underwent combined spinal-epidural anesthesia in the left lateral position. An epidural catheter was inserted at T12/L1 level, after which 5 mg of hyperbaric-bupivacaine and 15 μg of fentanyl were administered intrathecally at L3/4 intervertebral space. Additionally, 10 mL of 2% lidocaine (200 mg) was administered via the epidural catheter, allowing a T4 anesthetic intervertebral space to be attained. Figure 1 shows her vital sign trends during anesthesia, including cardiac index (CI), CVP, and ScvO_2_.

**Table 1:** Perioperative hemodynamic parameters

Status	Before anesthesia		After anesthesia induction		After delivery	
Oxygen (L/min)	None	5	5	5	5	5
ABP (s/d) (mm Hg)	132/65	137/73	109/53	122/60	124/59	117/52
HR (/min)	75	76	71	65	66	69
SaO_2_ (%)	92	98	97	95	95	95
ScvO_2_ (%)	52.2	54.6	38.6	42.1	67.3	58.7
CI (L/min/m^2^)	4.69	4.12	4.5	4.44	4.44	4.44
CO (L/min)	7.02	6.16	6.73	6.64	6.64	6.64
SV (mL)	98	85	100	107	106	101
CVP (mm Hg)	10	9	9	9	11	9
Hb (g/dL)	10.5	10.4	10.3	9.8	10.4	10.3
VO_2_ (mL/min)	393	373	543	461	256	333

VO_2_ (mL/min) = 1.34 × Hb (g/dL) × (SaO_2_ − ScvO_2_) × CO (L/min) × 10

*Abbreviations*: *ABP(s/d)* arterial blood pressure (systolic/diastolic), *CI* cardiac index, *CO* cardiac output, *CVP* central venous pressure, *Hb* hemoglobin, *HR* heart rate, *SaO*_*2*_ arterial oxygen saturation, *ScvO*_*2*_ central venous oxygen saturation, *SV* stroke volume, *VO*_*2*_ oxygen consumption

Ten minutes after the induction of anesthesia, she developed hypotension and her ScvO_2_ decreased to 38.6% compared to 54.6% before anesthesia. On the other hand, CO and CVP remained largely unchanged. Left uterine displacement was performed continuously after the induction of anesthesia. Additionally, phenylephrine was then infused intravenously at 0.5–1.5 mg/h. We selected not volume loading but vasoconstrictor for reversal of hypotension. Her ScvO_2_ levels recovered slightly with the increase of blood pressure. Nineteen minutes after induction, surgery was initiated. Ten minutes later, a healthy female infant weighing 2358 g was delivered. The infant’s Apgar scores at 1 and 5 min were 8 and 9, respectively. After delivery of the placenta, the patient’s ScvO_2_ increased from 45 to 83%, without blood pressure elevation; CVP increased from 10 to 14 mm Hg; and CI increased by 10%. VO_2_ values (Table 1) were calculated using the formula: VO_2_ (mL/min) = 1.34 × Hb (g/dL) × (SaO_2_ − ScvO_2_) × CO (L/min) × 10.

Surgery was completed without complications. For postoperative analgesia, 0.25% levobupivacaine was administered via the epidural catheter at 4 mL/h. Anesthesia and surgery lasted 69 and 50 min, respectively. During anesthesia, the estimated blood loss was 895 mL including amniotic fluid, and urine output was 220 mL. These were replaced with 750 mL of crystalloids.

The patient was transferred to the intensive care unit, where her CVP ranged from 11 to 15 mm Hg. Her brain natriuretic peptide levels increased by 14.2 to 26.7 pg/mL, although the cardiac silhouette on her chest X-ray did not show any dilation. For postoperative anticoagulation therapy, heparin was administered intravenously by 10,000 U/day. Her wound pain was well managed by epidural anesthesia. Heparin was stopped 6 h before the removal of the epidural catheter to prevent a hemorrhagic complication. She returned to the general ward on her first postoperative day and was discharged on the 9th postoperative day on 100 mg aspirin and 2.5 mg enalapril, daily.

## Discussion

Here, we reported a case describing the use of combined spinal-epidural anesthesia for the management of cesarean section in a Fontan-palliated parturient. Because some adult CHD patients are at high risk for developing heart failure during pregnancy and postpartum [11], we focused on monitoring hemodynamic changes by measuring ScvO_2_ (used to calculate VO_2_), APCO, and CVP.

During the perinatal period, intravascular volume increases and systemic vascular resistance substantially decreases, increasing cardiac output up to 7.0 L/min 12]. For a Fontan-palliated parturient, it is difficult for cardiac output (CO) to be adequately increased, making this type of aggressive volume challenge potentially dangerous for this population [12]. After the induction of spinal-epidural anesthesia, T4 anesthetic level was attained and blood pressure decreased below 100 mm Hg. Critical vasodilation and decreased preload were evident due to a sympathetic block [13]. To treat the hypotension, phenylephrine was administered rather than using aggressive volume loading because autotransfusion (approximately 500 mL), induced by uterine contraction after delivery, could potentially cause volume overload. Consequently, fluid balance during the cesarean section was only + 200 mL, although the patient’s CVP increased from 10 to 14 mm Hg without increasing CO. Postoperative diuretic administration decreased CVP to 11 mm Hg, and heart failure did not occur. Since Fontan-palliated patients have a reduced tolerance to volume overload, restrictive fluid therapy may be the most suitable option during cesarean section.

Our patient was born with TA, and her systemic ventricle was the morphologic left ventricle after Fontan procedure. Before pregnancy, her heart function and exercise capacity were preserved. Before anesthesia induction, her APCO was 7.02 L/min, which is equivalent to 140% of the CO of non-pregnant women and the CO of normal parturients [14]. While the ability to increase CO tends to be compromised in Fontan-palliated patients [12], our patient’s cardiac function fortunately allowed for CO increase. Meanwhile, her ScvO_2_ decreased to 52.2% as a result of a substantial VO_2_ increase. At the time, the VO_2_ level (393 mL/min; Table 1) was equivalent to 160% of the value expected for non-pregnant women at rest, and more than 140% of that of a normal parturient [14]. This sharp increase in VO_2_ may have been caused by maternal stress. Furthermore, the critically low ScvO_2_ levels observed indicate that she was at the limit of her cardiac capacity. If her systemic ventricle had been the morphologic right ventricle, she would probably have had significant systemic heart failure during the perinatal period.

After anesthesia induction, the patient’s arterial blood pressure slightly decreased to 109/53 mm Hg and CO was 6.73 L/min. ScvO_2_ levels further decreased to 38.6%, while VO_2_ levels further increased to 543 mL/min (Table 1). These changes in ScvO_2_ and VO_2_ levels may have resulted from further maternal psychological stress. After the delivery, her CO increased by 10% and CVP increased from 10 to 14 mm Hg. Additionally, her ScvO_2_ increased from 42.1 to 67.3% and the calculated VO_2_ decreased to 256 mL/min. The latter is equivalent to the VO_2_ of a non-pregnant woman at rest. This change in VO_2_ suggests that fetus delivery and subsequent stress relief may substantially decrease maternal VO_2_ and restore it to normal levels.

The combined spinal-epidural anesthesia management in a cesarean section for a Fontan-palliated parturient with monitoring of ScvO_2_, APCO, and CVP was presented. Our report revealed maternal circulatory dynamics and VO_2_ changes during a cesarean section in a Fontan-palliated patient. Maternal VO_2_ was found to decrease substantially after the delivery of the fetus.

## Data Availability

The datasets used and/or analyzed during the current study are available from the corresponding author on reasonable request.

## Abbreviations

CHD: Congenital heart disease
CVP: Central venous pressure
ScvO_2_: Central venous oxygen saturation
VO_2_: Oxygen consumption
APCO: Arterial pressure-based cardiac output
TA: Tricuspid atresia
NYHA: New York Heart Association
LVEF: Left ventricular ejection fraction
ABP: Arterial blood pressure
CI: Cardiac index
